# Prevalence and Risk Factors of Computer Vision Syndrome Among Bankers in Bangladesh

**DOI:** 10.1002/puh2.70259

**Published:** 2026-04-28

**Authors:** Bodrunnahar Barna, Md. Azad Uddin, Marzia Shormin, Md. Shihab Mostafa, Abdullah Al Islam

**Affiliations:** ^1^ Credit Information Bureau Bangladesh Bank, Head Office Dhaka Bangladesh; ^2^ Department of Statistics Shahjalal University of Science and Technology Sylhet Bangladesh

**Keywords:** Bangladesh, bank employees, computer vision syndrome, logistic regression, risk factors

## Abstract

**Background:**

The increasing reliance on digital devices in modern workplaces has raised significant concerns about computer vision syndrome (CVS), particularly among professionals in screen‐intensive environments, like the banking sector. Despite its widespread prevalence, limited research exists on CVS within the Bangladeshi banking sector. This study aims to estimate the prevalence of CVS among bank employees in Bangladesh and to evaluate the key demographic, occupational, and environmental risk factors associated with its occurrence.

**Methods:**

A cross‐sectional study was conducted using a structured, self‐administered questionnaire. The required sample size was calculated a prior based on an expected prevalence of 85.2%, a 5% margin of error, and a 95% confidence level, resulting in a target sample of 201 Bangladeshi bank employees. The presence of CVS was determined using the validated CVS‐Q tool. Descriptive statistics were conducted to summarize demographic and socioeconomic characteristics of the study participants. Logistic regression analysis was performed to identify significant predictors of CVS.

**Results:**

The prevalence of CVS among participants was 55.2%. The most reported symptoms were headache (79%), burning eyes (63%), and eye pain (52%). Logistic regression analysis revealed that increased daily computer use (odds ratios [OR]: 2.05; 95% CI: 1.07–4.83) and very bright monitor (OR: 113; 95% CI: 1.88–18,710) were significantly associated with higher odds of CVS. Conversely, larger family size (OR: 0.63; 95% CI: 0.39–0.92), higher weekly overtime (OR: 0.77; 95% CI: 0.60–0.91), and number of leave days taken for eye problems (OR: 0.03; 95% CI: 0.00–0.41) were associated with reduced odds of CVS.

**Conclusion:**

CVS is a prevalent occupational health concern among Bangladeshi bank employees, driven by modifiable factors such as screen brightness and prolonged computer use. Findings underscore the need for ergonomic interventions, regular eye screening, and educational measures to mitigate CVS and promote workplace well‐being.

## Graphical abstract

This graphical abstract illustrates the prevalence of computer vision syndrome and its associated demographic, occupational, and environmental risk factors among bank employees in Bangladesh.

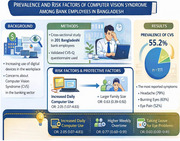

## 1 Introduction

The rapid advancement of technology has transformed workplaces globally, leading to a significant improvement in productivity and efficiency [1]. However, growing dependence on computers and other digital devices has led to some health concerns, particularly for professionals working in screen‐based industries. Studies show that prolonged screen time can cause vision problems, body pain, and stress [2, 3]. Visual effort is greater when viewing a computer screen compared to reading paper, primarily because the blink rate drops from 22 blinks per minute while reading paper to 7 blinks per minute, approximately during screen use. This reduced blinking causes increased tear evaporation and eye dryness [4]. Computer vision syndrome (CVS), also known as digital eye strain, refers to a collection of ocular and visual symptoms such as dryness, itching, burning eyes, double vision, or blurred vision resulting from prolonged use of digital screens like computers, tablets, and smartphones [5]. These symptoms, which may also include eye redness, a burning sensation, and other signs of eye strain, are typically short‐lived and resolve with rest, yet they can still negatively affect productivity and quality of life [6]. CVS has become the 21st century's most common occupational hazard, affecting more than 70% of all computer users and considered to be a global public health concern, as an estimated 60 million people suffer from CVS globally, and a million new CVS cases occur every year [7]. Studies have found several risk factors that might cause or worsen the symptoms of CVS, including brightness of the computer screen, brightness in the working area, continuous glaring of screen without blinking the eyes, duration of the computer use, poor sitting position, and not using blue light filtering eyeglasses [8, 9, 10, 11]. The prevalence of CVS is higher in developing countries than in developed countries because of limited access to and use of personal protective equipment, high workload, and limited break time during work [12]. Research revealed that the prevalence of CVS was 54.6% among call center operators in Brazil, 63% among university administrative workers in Malaysia, 67.4% among computer office workers in Sri Lanka, 67.8% in students of Lebanon and 69.3% among technology workers in Chennai [13, 14, 15, 16, 17]. In Africa, studies found that the prevalence of CVS was 65% in Nigeria and 51.5% in Ghana [13, 18]. Another study in Egypt showed that 92% of eye tiredness was reported among computer operators [19]. In Bangladesh, a cross‐sectional study was conducted in Bangabandhu Sheikh Mujib Medical University, which found the prevalence of CVS to be 46.8% [20].

Bank employees are particularly vulnerable as their job requires a lot of screen time for financial transactions, data processing, and customer service tasks [9]. Bankers are therefore susceptible to CVS as they have to engage in prolonged digital screen usage. A study conducted among bank employees in Peshawar, Pakistan, found that 77.2% of employees experienced CVS symptoms [21]. The prevalence of CVS among bank employees was reported as 74.6% in Addis Ababa, Ethiopia; 71.2% in Cape Coast Metropolis, Ghana; and 85.2% in Minia City, Egypt [22, 23, 24].

With the expansion of digital banking, financial technology (FinTech), and automated banking services, Bangladesh has witnessed a rapid transformation in its banking industry [25]. As a result, bankers are increasingly at risk of developing CVS in this country. There is a notable lack of empirical research on the prevalence and determinants of CVS among bank employees in Bangladesh. This study seeks to address this gap by assessing the prevalence of CVS among bank employees and identifying key demographic, occupational, and environmental risk factors.

## 2 Methodology

### 2.1 Study Design and Data Collection

A cross‐sectional study was conducted using a self‐administered structured questionnaire. The study included 201 participants, slightly exceeding the calculated minimum sample size of 194, which was based on an expected prevalence of 85.2% [24] with a margin of error of ±5% at 95% confidence. Participants were selected using a two‐stage sampling approach. Banks were first stratified into government (6 state owned commercial banks and 6 selected departments of the central bank, Bangladesh Bank) and private (43 private commercial banks and 9 foreign commercial banks) categories. From each stratum, a subset of banks and departments was randomly selected as 2 SOCBs and 3 Bangladesh Bank departments from the government stratum and 10 PCBs plus 3 FCBs from the private stratum. Only the head office of each selected bank was included, and employees within these offices were randomly invited to participate using a prepared list of email addresses. A slightly higher proportion of government employees was selected to ensure adequate representation. The questionnaire was prepared using Google Forms, and the link was sent to individuals working at different banks via email. The self‐administered questionnaire consists of four parts. The first part collects sociodemographic information of the participants. The second part assesses their pattern of computer use, collects information about a few variables that may influence the occurrence of CVS, and collects information regarding whether they wear protective eyeglasses or use monitor filters, whether they use adjustable chairs, take breaks, or do physical exercise regularly. The third part collects information about the frequency and intensity of sixteen symptoms of CVS. The last part collects information about medical expenditure and working hour loss due to the above‐mentioned health issue. Participants were also asked in the second part of the questionnaire whether they had any pre‐existing ocular diseases that could worsen symptoms of CVS. Ocular disease was considered clinically diagnosed eye conditions such as glaucoma, cataract, diabetic retinopathy, or chronic conjunctivitis.

#### 2.1.1 Inclusion Criteria

Full‐time bank employees, including officers, senior officers, and managerial staff who had been working for more than 1 year in any private or government bank and whose jobs required mandatory and regular use of a computer, were included in the study.

#### 2.1.2 Exclusion Criteria

Employees with a history of eye surgery or diagnosed serious eye diseases were excluded. Bank workers aged above 55 years and those whose duties were mainly outside the bank premises, like marketing officers, were also excluded from the study.

### 2.2 Study Variables

#### 2.2.1 Outcome Variable

The outcome variable in this study was the presence of CVS, which is a binary variable. To identify CVS in a participant, we used CVS‐Q [26], which is a validated standard questionnaire. The CVS‐Q questionnaire includes 16 different symptoms, including burning eyes, itching eyes, tearing eyes, feeling of a foreign body, excessive blinking, eye redness, eye pain, heavy eyelids, dryness of eyes, blurred vision, double vision, difficulty focusing for near vision, increased sensitivity to light, colored halos around objects, feeling that eyesight is worsening, and headaches. The frequency of the symptoms was categorized into three levels: never, occasionally, and often (coded as 0, 1, and 2, respectively). If the response is “occasionally” or “often,” the participant is further asked about the intensity of the symptoms, which can be moderate or intense (coded as moderate = 1 and intense = 2). The severity score for CVS is calculated by multiplying the frequency (0, 1, or 2) by the intensity scores (1 or 2) of these 16 symptoms and again assigning scores 0, 1, and 2 (0 = 0; 1, 2 = 1; 4 = 2) as severity scores. If the total severity score was equal to or greater than six, the subject was considered to suffer from CVS [27].

$$\begin{array}{ccc} \text{Severity}\;\text{score}\; & = & \;\sum (\text{frequency}\;\text{of}\;\text{symptom}\;\text{occurrence})\\ & & \times (\text{intensity}\;\text{of}\;\text{symptom}) \end{array}$$

The variable CVS was generated by the severity scores and coded as 0 and 1 (CVS = 1 when the severity score is ≥6, CVS = 0 otherwise).

#### 2.2.2 Explanatory Variables

Sociodemographic information of the participants, such as age, gender, marital status, and income, was assessed. Information on computer usage patterns included duration of service, daily computer use (hours), monitor and room brightness, viewing distance from the screen, screen level, and use of protective measures such as monitor filters, blue‐lightblocking glasses, eye drops, and adjustable chairs. Additional variables included the ability to adjust monitor brightness, break time during work, mandatory overtime, engagement in physical exercise, and type of computer used.

### 2.3 Statistical Analysis

Descriptive statistics were presented as mean ± standard deviations and median (range) for numerical variables and as percentages for categorical variables. A bar chart explaining the prevalence of the sixteen symptoms of CVS was prepared. A binary logistic regression analysis model was performed to detect the impact of demographic, occupational, and environmental factors on CVS. Odds ratios (OR) with 95% confidence intervals and corresponding *p* values were calculated to quantify the magnitude and direction of the effect of each predictor on the odds of developing the CVS outcome. Microsoft Excel 2016 was used for data cleaning and checking, and the data were analyzed with statistical software R version 4.3.1.

## 3 Results

### 3.1 Sociodemographic Characteristics

A total of 201 respondents participated in this study. Table 1 presents the descriptive statistics of different numeric variables assessed in the study. The mean age of participants was 31.5 (SD = 4.7) years. The average income of an individual was 53,415 taka (SD = 53,238), and the mean family size was 5.26 members (SD = 2.35). On average, participants had 5.6 years of service (SD = 4.9) and reported using a computer for 7.70 h per day (SD = 1.86). Furthermore, participants’ mean weekly overtime was 5.4 h (SD = 7.3). The average BMI for a participant was 24.22 (SD = 2.97). Additionally, participants reported an average of 19 min (SD = 28) of work distraction per day due to eye‐related issues.

**TABLE 1:** Descriptive statistics of numerical variables.

Variables	Mean (SD)	Median (range)
Age	31.5 (4.7)	30 (24–52)
Income	53,415 (53,238)	40,000 (12,000–680,000)
Family size	5.26 (2.35)	5 (1–15)
Service year	5.6 (4.9)	3.5 (1.0, 28.0)
Computer use per day	7.70 (1.86)	8.00 (3.00, 16.00)
Weekly overtime	5.4 (7.3)	3.0 (0.0, 55.0)
BMI	24.22 (2.97)	24.22 (16.40, 35.88)
Distracted from work due to eye problem (minutes)	19 (28)	10 (0, 180)

Abbreviation: SD, standard deviation.

Table 2 highlights the frequency distribution of various categorical variables among the respondents. Of the 201 participants, the majority were male (78%, *n* = 156), and most were married (66%, *n* = 132), whereas only 0.5% (*n* = 1) were divorced, widowed, or categorized as other. Ocular diseases were reported by 29% (*n* = 58) of respondents, and a small proportion (14%, *n* = 28) reported using monitor filters. Regarding screen settings, 64% (*n* = 128) of the respondents used monitors with medium brightness, whereas 32% of them used bright monitors. Moreover, room lighting was reported as bright by 53% (*n* = 107) of respondents. Over half of the participants (52%, *n* = 105) reported regularly adjusting their screen brightness. The distance between the eyes and the computer screen for most participants (72%, *n* = 145) was between 40 and 76 cm. 76% of the bank employees reported the level of their computer screen as at eye level. The majority (83%, *n* = 166) reported taking breaks during work, with 43% doing so every 2 h. Most of the respondents (94%, *n* = 188) did not use eye drops. Among those who did, 7% (*n* = 9) reported infrequent use. Blue light‐blocking eyeglasses were used by 28% (*n* = 57) of participants. Furthermore, almost half (46%, *n* = 93) of the respondents reported mandatory weekly overtime work. Exactly half of the respondents (50%, *n* = 101) engaged in regular physical exercise, and 73% (*n* = 147) reported having an adjustable chair at their workstations. Moreover, the vast majority (93%, *n* = 187) used a desktop computer. Regarding sleep issues, 24% (*n* = 48) indicated that they often had trouble falling asleep. CVS was reported by 55.2% (*n* = 111) of the participants. The majority (67%, *n* = 134) worked in the private sector, and 61% (*n* = 123) reported feeling frequent stress.

**TABLE 2:** Frequency table of categorical variables.

	Study participants *n* = 201	
Characteristics	*N*	%
Gender		
Female	45	22
Male	156	78
Marital status		
Divorced/Widowed/Others	1	0.5
Married	132	65.5
Unmarried	68	34
Ocular disease		
No	143	71
Yes	58	29
Monitor filters		
No	173	86
Yes	28	14
Monitor brightness		
Bright	65	32
Dull	2	1.0
Medium	128	64
Very bright	6	3.0
Room brightness		
Bright	107	53
Dark	4	2.0
Medium	73	36
Very bright	17	9.0
Adjust computer brightness		
No	96	48
Yes	105	52
Distance between eyes and screen		
Between 40 and 76 cm (about an arm's length away)	145	72
Less than 40 cm (less than an arm's length away)	37	18
More than 76 cm (more than an arm's length away)	19	10
Level of computer screen		
Above the level of eyes	18	9.0
At the level of eyes	152	76
Below the level of eyes	31	15
Breaks during work		
No	35	17
Yes	166	83
Break interval		
Every 30 min	24	12
Every hour	27	13
Every 2 h	86	43
More	64	32
Eye drops		
No	188	93.5
Yes	13	6.5
Frequency of using eye drops		
Always	2	1.6
Never	118	87
Rarely	6	4.5
Sometimes	9	6.9
Blue light‐blocking eyeglass		
No	144	72
Yes	57	28
If weekly overtime mandatory		
No	108	54
Yes	93	46
Exercise		
No	100	49.8
Yes	101	50.2
Adjustable chair		
No	54	26.9
Yes	147	73.1
Computer type		
Desktop	187	93
Laptop	13	6.5
Other	1	0.5
Trouble going to sleep		
Never	36	18
Often	48	24
Rarely	49	24
Sometimes	68	34
CVS		
No	90	44.8
Yes	111	55.2
Sector type		
Govt.	66	33
Private	134	67
Stress		
No	78	38.8
Yes	123	61.2

**FIGURE 1:**
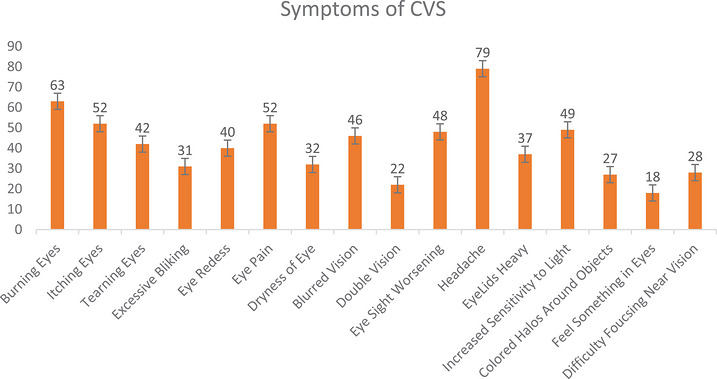
Distribution of symptoms of computer vision syndrome (CVS) among study participants.

Figure 1 illustrates the distribution of reported symptoms of CVS among the study participants, expressed as percentages. The most frequently reported symptom was headache, affecting 79% of respondents, followed by burning eyes (63%) and itching eyes (52%). Other common symptoms included eye pain (52%), increased sensitivity to light (49%), blurred vision (46%), tearing eyes (42%), and eye redness (40%). Less frequently reported symptoms included dryness of the eye (32%), excessive blinking (31%), difficulty focusing on near vision (28%), colored halos around objects (27%), and double vision (22%). The least reported symptom was feeling something in the eyes, noted by only 18% of respondents.

### 3.2 Findings From the Logistic Regression Analysis

Table 3 presents the results of the binary logistic regression model, reporting OR with 95% confidence intervals for identifying various risk factors significantly associated with the occurrence of CVS at the 5% level of significance. Statistically significant associations were observed for family size (*p* = 0.030), daily computer use (*p* = 0.033), very bright monitor (*p* = 0.043), weekly overtime (*p* = 0.010), income (*p* = 0.048), and number of leave days taken for eye problems (*p* = 0.020). Detailed logistic regression outputs are provided in Table S1. The results highlighted that for each additional unit increase in family size, the odds of having CVS decreased by 37% (OR: 0.63; 95% CI: 0.39–0.92). Moreover, for each additional unit increase in computer use per day, the odds of having CVS increased by 2.05 (OR: 2.05; 95% CI: 1.07–4.83) times. Employees using monitors set to very high brightness levels had markedly higher odds of CVS (OR: 113; 95% CI: 1.88–18,710) compared to those using normal brightness settings. However, this estimate is accompanied by an extremely wide confidence interval, indicating considerable uncertainty, likely due to the small number of participants in this category. Each additional unit increase in weekly overtime, the odds of having CVS were observed to decrease by 23% (OR: 0.77; 95% CI: 0.60–0.91); however, this finding should be interpreted cautiously, whereas per unit increase of total cost for eye‐related and income showed a statistically significant but practically negligible effect on CVS likelihood (OR: 1.00). Additionally, for each additional leave day taken for eye problems, risk of having CVS decreases by 97% (OR: 0.03; 95% CI: 0.00–0.41), a strong negative association was observed; however, this finding may reflect behavioral or temporal factors. Finally, if respondents had thyroid problems, the risk of having CVS was almost zero (OR: 0.00) compared to those who had no thyroid problems; an extremely low estimated odds ratio was observed, likely influenced by sparse data. Overall, these findings should be interpreted as exploratory due to potential instability in some estimates.

**TABLE 3:** Results of binary logistic regression of computer vision syndrome (CVS).

Risk factors	Odds ratio (95% CI)
Family size	0.63 (0.39–0.92)^*^
Computer use per day	2.05 (1.07–4.83)^*^
Monitor brightness	
Normal bright	—
Very bright	113 (1.88–18,710)^*^
Weekly overtime	0.77 (0.60–0.91)^*^
Total cost for eye‐related treatment	1.00 (1.00–1.00)^*^
Income	1.00 (1.00–1.00)^*^
Number of leaves for eye problem	0.03 (0.00–0.41)^*^
Thyroid	
No	—
Yes	0.00 (0.00–0.00)^*^

* *p* value < 0.05.

### 3.3 Receiver Operating Characteristic (ROC) Curve Analysis

**FIGURE 2:**
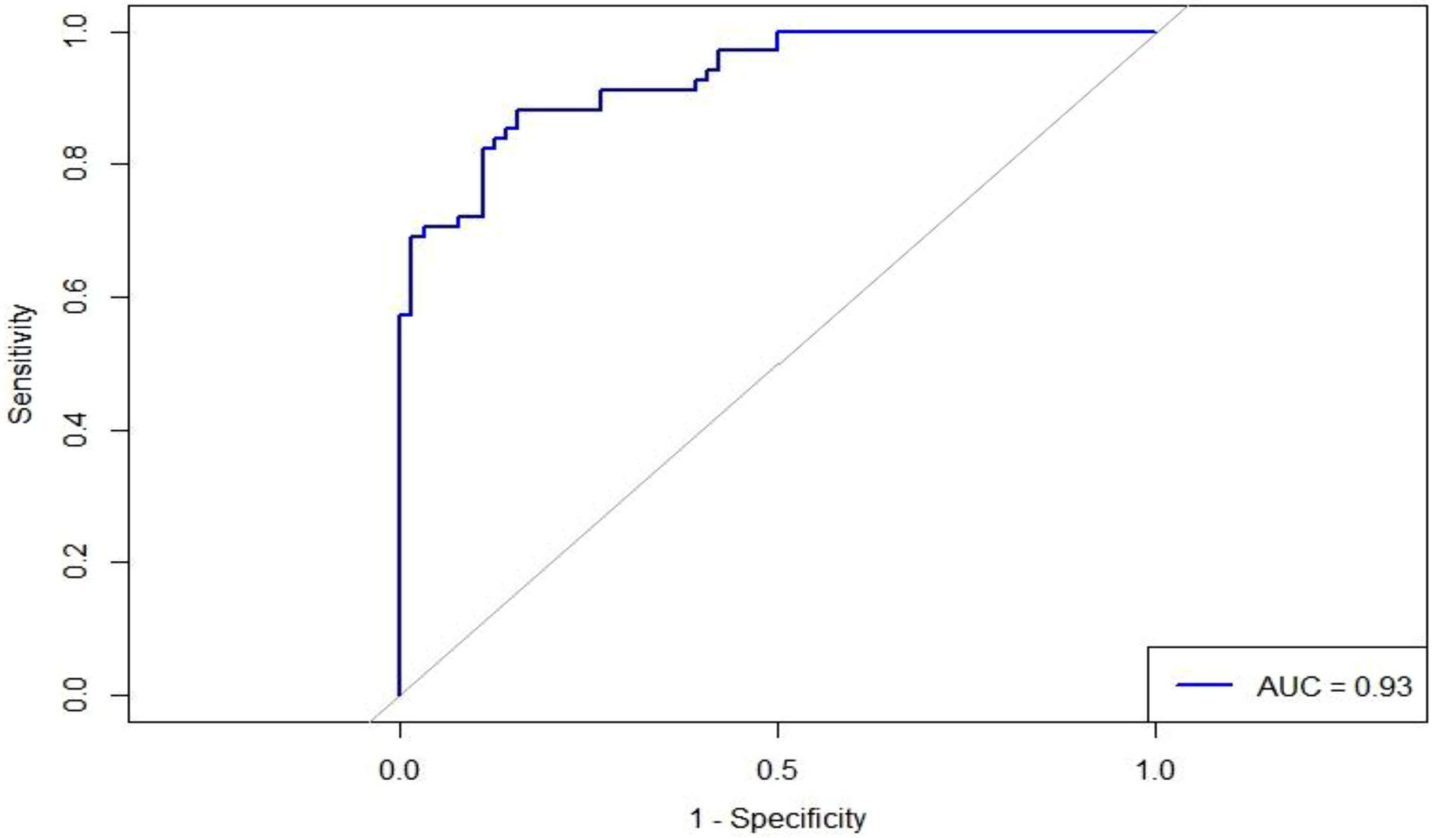
ROC curve of logistic regression model for predicting computer vision syndrome (CVS). AUC, area under the curve.

The logistic regression model's performance in predicting CVS was evaluated using an ROC curve (Figure 2). The model showed high apparent discriminative ability, with an area under the curve (AUC) of 0.93. This indicates that the model can reliably distinguish between individuals with and without CVS, highlighting the strength of the included predictors in identifying those at risk. However, no internal validation (e.g., cross‐validation or bootstrapping) was performed; therefore, the reported AUC may be optimistic and should be interpreted with caution.

## 4 Discussion

This study aimed to explore the prevalence of CVS as well as its demographic, occupational, and environmental contributors, among bank employees in Bangladesh who use digital screens for an extended period. The prevalence of CVS among bank employees in our study was 55.2%. However, previous studies have demonstrated a higher prevalence of CVS, 77.2%, among bank employees in Peshawar, Pakistan [21], and 74.6% among commercial bank employees in Addis Ababa, Ethiopia [23]. The higher prevalence reported in the Peshawar study may be due to considering the presence of one or more symptoms for a CVS diagnosis. In the Addis Ababa study, CVS was defined as having at least one of ten symptoms, either intermittently or continuously, over the past 12 months in one or both eyes. The higher prevalence reported in studies may be due to the broader diagnostic criteria. In contrast, our study used a more detailed approach, assessing 16 symptoms with frequency and intensity, which may explain the lower prevalence observed.

The most common reported symptom was headache (79%), followed by burning eyes (63%), eye itching (52%), and eye pain (52%), consistent with earlier studies that describe these as typical manifestations of CVS [28]. Ranasinghe reported that in their study, headache (45.7%), dry eyes (31.1%), and pain in and around the eyes (28.7%) were the most prevalent symptoms of CVS [7]. Headache was the most common symptom reported in several similar studies [29, 30, 31]. The high prevalence of headache observed in the present study may be attributed to accommodative spasm associated with sustained near work and prolonged computer use, particularly among younger participants [32].

The logistic regression analysis revealed several significant associations. However, some estimated ORs were large and accompanied by wide confidence intervals, particularly for categories with small sample sizes. Increased daily computer usage and higher monitor brightness were significantly associated with higher odds of developing CVS. These findings support prior research, which suggests that prolonged use of computer screens and inappropriate screen luminance can worsen CVS and ocular discomfort [7, 33]. However, some estimates, particularly for very high monitor brightness, were large and accompanied by a wide confidence interval, indicating a degree of uncertainty, likely influenced by small sample sizes in certain categories. Some associations observed in this study appeared counterintuitive. For example, weekly overtime and the number of leave days taken for eye‐related problems were negatively associated with CVS. These findings are unlikely to represent true protective effects and may instead reflect reverse causation, where individuals experiencing symptoms modify their behavior (e.g., reducing screen exposure or taking leave). Behavioral adaptation and residual confounding may also explain these relationships. Similarly, the negative association with family size may be influenced by unmeasured lifestyle or occupational factors rather than a direct effect. Additionally, individuals reported an average of 19 min of daily work distraction caused by eye‐related symptoms. This finding highlights the substantial impact that visual discomfort associated with CVS can have on workplace efficiency and the overall productivity of employees. Overall, the multivariable analysis provides exploratory insights, and the observed associations should be considered hypothesis‐generating rather than confirmatory.

Furthermore, the high discriminative ability of the model (AUC = 0.93) should be interpreted with caution, as no internal validation (e.g., cross‐validation or bootstrapping) was performed. As a result, the reported performance may be optimistic and could reflect overfitting. Future studies should incorporate validation techniques and larger samples to confirm these findings.

## 5 Conclusion

This study provides valuable insights into the prevalence of CVS among bank employees working with digital screens. More than half of the respondents reported symptoms related to CVS, underscoring a growing public health concern, especially in the banking sector, where employees are required to engage in prolonged computer use as part of their routine tasks. The findings emphasize the role of modifiable risk factors like monitor brightness, daily computer usage, and workplace ergonomics. Overall, these findings underscore the need for targeted awareness programs, ergonomic interventions, and regular eye check‐ups to prevent and manage CVS effectively. In Bangladesh, the scarcity of research on CVS within the banking sector despite the sector's intensive screen‐based work underscores the urgent need for comprehensive studies to inform evidence‐based prevention and intervention strategies.

## 6 Limitations of the Study

This study has several limitations. The assessment of CVS was based on a self‐reported questionnaire, and the subjective nature of the data may introduce reporting bias. In addition, objective ophthalmic measurements such as tear break‐up time, Schirmer test, and refractive assessment to evaluate accommodative spasm were not performed. Future studies incorporating objective clinical examinations alongside subjective assessments are warranted to provide a more comprehensive evaluation of CVS.

## Author Contributions

**Bodrunnahar Barna**: conceptualization, data curation, formal analysis, investigation, methodology, resources, software, validation, visualization, writing – original draft, writing – review and editing. **Md. Azad Uddin**: methodology, software, validation, visualization, writing – review and editing. **Marzia Shormin**: methodology, software, validation, visualization, writing – review and editing. **Md. Shihab Mostafa**: methodology, software, validation, visualization, writing – review and editing. **Abdullah Al Islam**: conceptualization, methodology, resources, software, supervision, validation, visualization, writing – original draft, writing – review and editing.

## Funding

The authors have nothing to report.

## Ethics Statement

This study involved a cross‐sectional, anonymous survey of adult banking professionals, focusing on sociodemographic characteristics, workplace conditions, and self‐reported symptoms associated with computer vision syndrome (CVS). No clinical procedures were performed, and no sensitive or personally identifiable information was collected. Participation was entirely voluntary, and informed consent was obtained from all participants after they were informed about the study's purpose, the confidentiality of their responses, and their right to decline or withdraw at any time without consequence. Our study fully complies with the ethical principles of the Declaration of Helsinki and established standards for minimal‐risk research involving human participants. As this study is observational in nature and does not involve a clinical trial, formal trial registration was not required.

## Conflicts of Interest

The authors declare no conflicts of interest.

## Transparency Statement

The corresponding author, “Abdullah Al Islam,” affirms that this manuscript is an honest, accurate, and transparent account of the study being reported; that no important aspects of the study have been omitted; and that any discrepancies from the study as planned (and, if relevant, registered) have been explained.

## Supporting information




**Supporting file 1**: puh270259‐sup‐0001‐Table.docx

## Data Availability

Upon a reasonable request, data would be made available.
