# Supplementary material for: Prevalence and Risk Factors of Computer Vision Syndrome Among Bankers in Bangladesh
Source: Public Health Chall. 2026 Apr 28;5(2):e70259. doi: 10.1002/puh2.70259 (PMC13123453; doi:10.1002/puh2.70259)
Supplement: Supplementary file 1 — Supporting file 1: puh270259‐sup‐0001‐Table.docx [file PUH2-5-e70259-s001.docx]

**Supplementary Table 1** Logistic Regression Model for CVS

| Characteristic | OR*^1^* | p-value |
| --- | --- | --- |
| Age | 0.86 | 0.5 |
| Gender |  |  |
| Female | — |  |
| Male | 0.61 | 0.7 |
| Marital Status |  |  |
| Married | — |  |
| Unmarried | 2.74 | 0.3 |
| Income | 1.00 | **0.048** |
| Family size | 0.63 | **0.030** |
| Ocular Disease |  |  |
| No | — |  |
| Yes | 3.45 | 0.3 |
| Service year | 1.29 | 0.081 |
| Computer use per day | 2.05 | **0.033** |
| Monitor Filters |  |  |
| No | — |  |
| Yes | 3.02 | 0.4 |
| Monitor Brightness |  |  |
| Normal bright | — |  |
| Medium bright | 5.74 | 0.12 |
| Very bright | 113 | **0.043** |
| Room Brightness |  |  |
| Bright | — |  |
| Dark | 17.5 | 0.3 |
| Medium | 1.75 | 0.6 |
| Very bright | 9.47 | 0.14 |
| Adjust Computer Brightness |  |  |
| No | — |  |
| Yes | 0.72 | 0.7 |
| Distance Between Eyes and screen |  |  |
| Between 40 and 76 cm (about an arm’s length away) | — |  |
| Less than 40 cm (less than an arm’s length away) | 2.58 | 0.4 |
| More than 76 cm (more than an arm’s length away) | 0.10 | 0.13 |
| Level of Computer Screen |  |  |
| Above the level of eyes | — |  |
| At the level of eyes | 0.26 | 0.3 |
| Below the level of eyes | 0.89 | >0.9 |
| Breaks during work |  |  |
| No | — |  |
| Yes | 4.33 | 0.2 |
| Break Interval |  |  |
| Every 30 min | — |  |
| Every hour | 0.50 | 0.7 |
| Every two hours | 2.27 | 0.7 |
| More | 3.33 | 0.5 |
| Eye drops |  |  |
| No | — |  |
| Yes | 0.03 | 0.4 |
| Frequency of using Eye drops |  |  |
| Always | — |  |
| Never | 0.00 | >0.9 |
| Rarely | 0.00 | >0.9 |
| Sometimes | 0.01 | >0.9 |
| Blue Light blocking eye glass |  |  |
| No | — |  |
| Yes | 0.21 | 0.2 |
| Weekly overtime | 0.77 | **0.010** |
| If weekly overtime mandatory |  |  |
| No | — |  |
| Yes | 1.26 | 0.8 |
| Exercise |  |  |
| No | — |  |
| Yes | 0.34 | 0.3 |
| Adjustable Chair |  |  |
| No | — |  |
| Yes | 2.03 | 0.4 |
| Trouble going to sleep |  |  |
| Never | — |  |
| Often | 4.42 | 0.4 |
| Rarely | 0.06 | 0.11 |
| Sometimes | 0.10 | 0.2 |
| Back Pain |  |  |
| No | — |  |
| Yes | 8.41 | 0.11 |
| Pain in hands/wrists/arms/shoulders |  |  |
| No | — |  |
| Yes | 0.85 | 0.9 |
| BMI | 1.22 | 0.2 |
| Sector Type |  |  |
| Govt. | — |  |
| Private | 0.10 | 0.072 |
| Stress |  |  |
| No | — |  |
| Yes | 1.80 | 0.5 |
| Total Cost for Backpain Treatment | 1.00 | 0.2 |
| Total Cost for Eye Related Treatment | 1.00 | **0.019** |
| Distracted From Work Due to Backpain (Minutes) | 0.97 | 0.3 |
| Distracted From Work Due to Eye Problem (Minutes) | 1.03 | 0.3 |
| Number of Leaves for Eye Problem | 0.03 | **0.020** |
| Allergy |  |  |
| No | — |  |
| Yes | 2.36 | 0.5 |
| Rheumatoid Arthritis |  |  |
| No | — |  |
| Yes | 235 | >0.9 |
| Diabetes |  |  |
| No | — |  |
| Yes | 0.33 | 0.7 |
| Thyroid |  |  |
| No | — |  |
| Yes | 0.00 | **0.032** |
| Others |  |  |
| No | — |  |
| Yes | 7.84 | 0.2 |
| *^1^*OR = Odds Ratio | | |
